# Topological Dissection of Proteomic Changes Linked to the Limbic Stage of Alzheimer’s Disease

**DOI:** 10.3389/fimmu.2021.750665

**Published:** 2021-10-12

**Authors:** Erika Velásquez, Beáta Szeitz, Jeovanis Gil, Jimmy Rodriguez, Miklós Palkovits, Éva Renner, Tibor Hortobágyi, Péter Döme, Fábio CS. Nogueira, György Marko-Varga, Gilberto B. Domont, Melinda Rezeli

**Affiliations:** ^1^ Section for Clinical Chemistry, Department of Translational Medicine, Lund University, Skåne University Hospital Malmö, Malmö, Sweden; ^2^ Division of Oncology, Department of Internal Medicine and Oncology, Semmelweis University, Budapest, Hungary; ^3^ Division of Oncology, Department of Clinical Sciences Lund, Lund University, Lund, Sweden; ^4^ Division of Chemistry I, Department of Medical Biochemistry and Biophysics, Karolinska Institute, Stockholm, Sweden; ^5^ Human Brain Tissue Bank, Semmelweis University, Budapest, Hungary; ^6^ Institute of Pathology, Faculty of Medicine, University of Szeged, Szeged, Hungary; ^7^ ELKH-DE Cerebrovascular and Neurodegenerative Research Group, Department of Neurology, Faculty of Medicine, University of Debrecen, Debrecen, Hungary; ^8^ Department of Psychiatry and Psychotherapy, Semmelweis University, Budapest, Hungary; ^9^ National Institute of Mental Health, Neurology and Neurosurgery, Budapest, Hungary; ^10^ Proteomics Unit, Department of Biochemistry, Institute of Chemistry, Federal University of Rio de Janeiro, Rio de Janeiro, Brazil; ^11^ Laboratory of Proteomics, Laboratório de Apoio ao Desenvolvimento Tecnológico (LADETEC), Institute of Chemistry, Federal University of Rio de Janeiro, Rio de Janeiro, Brazil; ^12^ Division of Clinical Protein Science & Imaging, Department of Biomedical Engineering, Lund University, Lund, Sweden

**Keywords:** Alzheimer’s disease, limbic stage, proteomics, phosphoproteomics, acetylomics, neuroinflammation

## Abstract

Alzheimer’s disease (AD) is a neurodegenerative disorder and the most common cause of dementia worldwide. In AD, neurodegeneration spreads throughout different areas of the central nervous system (CNS) in a gradual and predictable pattern, causing progressive memory decline and cognitive impairment. Deposition of neurofibrillary tangles (NFTs) in specific CNS regions correlates with the severity of AD and constitutes the basis for disease classification into different Braak stages (I-VI). Early clinical symptoms are typically associated with stages III-IV (i.e., limbic stages) when the involvement of the hippocampus begins. Histopathological changes in AD have been linked to brain proteome alterations, including aberrant posttranslational modifications (PTMs) such as the hyperphosphorylation of Tau. Most proteomic studies to date have focused on AD progression across different stages of the disease, by targeting one specific brain area at a time. However, in AD vulnerable regions, stage-specific proteomic alterations, including changes in PTM status occur in parallel and remain poorly characterized. Here, we conducted proteomic, phosphoproteomic, and acetylomic analyses of human postmortem tissue samples from AD (Braak stage III-IV, n=11) and control brains (n=12), covering all anatomical areas affected during the limbic stage of the disease (total hippocampus, CA1, entorhinal and perirhinal cortices). Overall, ~6000 proteins, ~9000 unique phosphopeptides and 221 acetylated peptides were accurately quantified across all tissues. Our results reveal significant proteome changes in AD brains compared to controls. Among others, we have observed the dysregulation of pathways related to the adaptive and innate immune responses, including several altered antimicrobial peptides (AMPs). Notably, some of these changes were restricted to specific anatomical areas, while others altered according to disease progression across the regions studied. Our data highlights the molecular heterogeneity of AD and the relevance of neuroinflammation as a major player in AD pathology. Data are available *via* ProteomeXchange with identifier PXD027173.

## 1 Introduction

Alzheimer’s disease (AD) is a severe neurodegenerative disorder and the most common cause of dementia worldwide. Early-onset AD is a rare hereditary form of the disease that accounts for ~5-10% of all cases and a minority of early-onset cases has been linked to specific mutations in the genes encoding amyloid precursor protein (APP), presenilin 1 (PSEN1), and presenilin 2 (PSEN2) (1, 2). Late-onset or sporadic AD has both genetic (related mainly to apolipoprotein E gene variants) and environmental components and has been associated with multiple risk factors, including age and gender (1, 3). Histologically, AD is characterized by *inter alia* the extracellular deposition of amyloid-β (Aβ) plaques and the intraneuronal accumulation of neurofibrillary tangles (NFTs), which correlates with progressive cognitive decline (4). Despite current evidence of direct involvement of Aβ and NFTs in the pathology of AD, the molecular basis of the disease remains unclear (5).

Recent findings suggest that neuroinflammation might be a central mechanism linking Aβ pathology and NFTs development to AD progression (6). Indeed, clinical studies suggest that neuroinflammation often precedes plaque deposition and NFT accumulation, which in turn triggers a chronic inflammatory condition that exacerbates more amyloid deposition and cognitive dysfunction (6, 7). Further support for the critical role of immune activation in AD development comes from genome-wide association studies (GWAS), and the identification of multiple AD risk genes linked to innate immunity and inflammation (8). *In vivo* and *in vitro* studies have shown that microglia activation *via* the triggering receptor expressed on myeloid cells-2 (TREM2) triggers the release of cytokines such as TNF-α, IL-1β, IL-6, and IL-8 and the modulation of astrocyte functions, which results in synaptic loss and neuronal damage (9). However, the impact of these inflammatory changes on the progression of AD pathology across vulnerable brain regions is poorly understood.

The development of neurodegenerative changes in AD follows a regular pattern, revealing the selective vulnerability of certain brain areas to disease progression. Region-specific accumulation of NFTs correlates with disease severity (10). For example, NFTs during the preclinical phase of AD (stage I-II) are often limited to the transentorhinal (anatomically described as the perirhinal cortex) and entorhinal cortices (EC), and the CA1 region, whereas NFT spread to the hippocampus is associated with the limbic stages of the disease (stages III-IV), when early clinical symptoms appear. Lastly, the involvement of neocortical areas (stages V-VI) is associated with more severe histopathological changes, cognitive impairment, and memory decline (10, 11). The molecular basis for this regional vulnerability has not been fully elucidated. Regional heterogeneity in the morphology and transcriptional profiles of specific neurons and glial cells subpopulations therein have been associated with the vulnerability of early AD-affected brain regions (12, 13). It is also possible that local differences in the brain proteome might shape the AD vulnerability of different regions.

Recently, proteomics has emerged as a valuable tool to examine large-scale proteome alterations in brain tissue and cerebrospinal fluid during AD progression, revealing profound changes in pathways that regulate oxidative phosphorylation and synaptic function in early *vs* late AD stages (14). Alterations in lipid metabolism, iron homeostasis, membrane transport, WNT signaling, and mRNA processing have also been reported (15–18). Most notably, the proteomic analysis also revealed dysregulation of inflammatory pathways in AD, including changes in microglia and astrocyte activation (19, 20). Unfortunately, most proteomic studies to date focusing on AD progression usually target one single brain area at a time. Stage-specific proteomic alterations of AD across multiple vulnerable regions remain poorly characterized, including changes in posttranslational modifications (PTMs), such as phosphorylation and acetylation. Finding proteome signatures of the progression of AD across specific brain areas might help to identify novel pathways that can be targeted to prevent regional neurodegeneration, and to preserve neuronal functions. Here, we conducted a topological dissection of AD brains through an integrated multi-omics approach (proteomics, phosphoproteomics, and acetylomics) on human postmortem tissue from clinically important limbic stages of the disease. Our data highlights the molecular heterogeneity of AD across brain regions and the importance of neuroinflammation as a major player in AD pathology.

## 2 Methods

### 2.1 Sample Cohort

Postmortem brain tissues derived from the hippocampus, CA1 region of the hippocampal cortex (21), entorhinal (Brodmann area 34) and perirhinal cortices (Brodmann area 35) (22) were dissected out by the micropunch technique as previously described (23). The histopathological analysis and disease staging were based on the presence of the NFTs, neuropil threads, and neuritic plaques, following standardized criteria as previously described (24). A total of 56 tissue samples from eleven AD (Braak stage III-IV) and twelve control brains were analyzed (**Table S1**). All brain samples were obtained from the Human Brain Tissue Bank (Semmelweis University, Budapest, Hungary). Human brain microdissection procedures were approved by the Regional and Institutional Committee of Science and Research Ethics of Scientific Council of Health (34/2002/TUKEB-13716/2013/EHR, Budapest, Hungary) and the Code of Ethics of the World Medical Association (Declaration of Helsinki).

### 2.2 Protein Extraction

The frozen tissue samples were sectioned using a cryostat (LEICA CM1950), and ten to twenty 10 μm slices were collected in Eppendorf tubes for proteomic analysis. Samples were resuspended and vortexed in 100-300 μL of protein extraction buffer (50 mM dithiothreitol (DTT), 2% sodium dodecyl sulfate (SDS), 100 mM tris(hydroxymethyl)aminomethane hydrochloride (Tris-HCl) pH 8.6). The lysates were incubated at 95°C for 5 min with constant shaking at 500 rpm (Thermomixer Comfort, Eppendorf AG), and then sonicated in the Bioruptor Plus UCD-300 (Diagenode) (40 cycles, 15s on/off) followed by centrifugation at 20,000 g for 20 min at 18°C (Thermo Scientific Sorvall ST 8R Centrifuge). The supernatants were collected and transferred to new tubes. The proteins were alkylated with 100 mM iodoacetamide for 30 min in the dark at room temperature (RT), and then precipitated using 9 volumes of cold ethanol overnight at -20°C. The pellet was washed three times with 1 mL of 90% ethanol, then dried at room temperature and preserved at -20°C until further use.

### 2.3 Chemical Acetylation and Protein Digestion for Global Proteome and Acetylome Analysis

The protein pellet was dissolved in a solution of 1% SDS, 0.5% sodium deoxycholate (SDC), 100 mM tetraethylammonium bromide (TEAB) (pH 8.0). For acetylation stoichiometry analysis, the reagent N-acetoxy-succinimide-d3 (NAS-d3) was synthesized as described previously (25–27). The unmodified lysine residues were chemically acetylated by incubation with a 100-fold molar excess of NAS-d3 in dimethyl sulfoxide at RT for 1h. This labeling step was repeated once more. The samples were finally incubated with 5% hydroxylamine for 20 min to revert unspecific reactions such as the O-acetylation of hydroxylated amino acids. The samples were precipitated using cold ethanol as described before to remove the excess of reagents and then resuspended in 0.5% SDC, 50 mM ammonium bicarbonate (pH 8.0). Protein amounts were estimated using the BCA protein determination assay (BCA™ Protein Assay Kit).

The proteins were digested using trypsin (enzyme: substrate, 1:50) at 37°C overnight (Sequencing Grade Modified Trypsin, Promega, Madison, WI, USA). After trypsinization the peptides were cleaned up using ethyl-acetate extraction (1:1, samples/solvent) under acidic conditions (final concentration of trifluoroacetic acid (TFA) was 0.5%). The organic phase was discarded, and an additional cleaning step was conducted using ethyl acetate (1:1). The organic phase was discarded, and peptides were dried in a speed-vac for 5 min to evaporate the remaining solvent. The peptides were resuspended in 2% acetonitrile (ACN) with 0.1% TFA and peptide determination was performed using the Pierce™ Quantitative Colorimetric Peptide Assay. The samples were used for global proteome and acetylome analyses.

### 2.4 Protein Digestion and Phosphopeptide Enrichment for Phosphoproteome Analysis

For phosphoproteome analysis, the protein pellet was dissolved in a solution of 0.5% SDC, 100 mM ammonium bicarbonate (pH 8.0), and the proteins were digested using trypsin (enzyme: substrate, 1:50) at 37°C overnight. After digestion, the peptides were cleaned up using ethyl-acetate extraction as described before. The peptides were resuspended in 40 μL of 0.1% TFA and peptide concentration was determined using the Pierce™ Quantitative Colorimetric Peptide Assay. For phosphopeptide enrichment, 55 µg of peptides were submitted to an automatic workflow on the AssayMAP Bravo system (Agilent Technologies) using the Fe(III)-IMAC assay as previously described (Murillo et al, 2018). The phosphopeptides were resuspended in 2% ACN with 0.1% TFA before nLC-MS/MS analysis.

### 2.5 LC-MS/MS Analysis

One microgram of the peptides was injected for each global proteome analysis and the total amount of enriched peptides was used for phosphoproteome analysis. The samples were analyzed on a Q-Exactive HF-X mass spectrometer coupled to an UltiMate 3000 RSLCnano system (Thermo Scientific). Peptides were concentrated on an Acclaim PepMap™ 100 (75 μm × 2 cm, nanoViper) trap-column and separated on a PepMap RSLC C18 (2 μm, 100 Å, 75 μm x 25 cm) analytical column. The nanoLC system was operated at a flow rate of 300 nL/min and the column temperature was set to 45°C. The solvents used for the nonlinear gradient were A (0.1% formic acid) and B (0.08% formic acid in 80% ACN).

For total proteome and acetylome analysis, the gradient started with 2% solvent B and increased to 27% during 112 min, in the next 10 min solvent B increased to 35%, then to 50% in 7 min and finally, it augmented to 90% in 8 min and it was kept at 90% for 5 min. Samples were analyzed using a top 20 data-dependent acquisition (DDA) method, the spray voltage was set to 1.85 kV. MS1 full scans were acquired with 120,000 (@ 200 m/z) resolution, target AGC value of 3e6, and maximum injection time of 100 ms. For MS2 analysis the 20 most intense ions were selected for higher-energy collisional dissociation (HCD) with an NCE of 28. MS2 spectra were acquired with 15,000 (@ 200 m/z) resolution, target AGC value of 1e5, and maximum injection time of 50 ms. The ion selection threshold was set to 1.6e5 and the dynamic exclusion to 40 s.

The phosphoproteome analysis was performed using a gradient starting with 4% solvent B and increased to 27% during 120 min, in the next 15 min solvent B increased to 45% and finally, it augmented to 98% in 1 min and it was kept at 98% for 5 min. Samples were analyzed using a top 15 DDA method, the spray voltage was set to 1.85 kV. MS1 full scans were acquired with 120,000 resolution, target AGC value of 3e6, and maximum injection time of 50 ms. For MS2 analysis the 15 most intense ions were selected for fragmentation with an NCE of 25. MS2 spectra were acquired with 60,000 resolution, target AGC value of 1e5, and maximum injection time of 120 ms. The ion selection threshold was set to 7.2e3 and the dynamic exclusion to 30 s.

### 2.6 Database Searching

Raw files from global proteomics were analyzed in Proteome Discoverer 2.2 software environment (Thermo Scientific) using SEQUEST HT as search engine against the UniProtKB human database (version from 01/15/2019, canonical and isoforms, 42,356 sequences). The following search parameters were employed: Arg-C as the cleavage enzyme, carbamidomethylation at cysteine residues as a static modification, methionine oxidation, acetylation [normal (d0) and heavy (d3)] at lysine residues, and at protein N-terminus as dynamic modifications, 10 ppm tolerance for precursor ions and 0.02 Da for fragment ions. A maximum of 2 missed cleavages were allowed and the minimum peptide length was set to six amino acids. The filter High Confidence (FDR q value = 0.01) was applied on both peptide and protein levels.

For the phosphoproteome analysis, the same software, protein database, and search engine were used with the following search parameters: Trypsin as the cleavage enzyme, carbamidomethylation at cysteine residues as a static modification, phosphorylation of serine, threonine and tyrosine, oxidation of methionine and acetylation at protein N-terminus as dynamic modifications, 10 ppm tolerance for precursor ions and 0.02 Da for fragment ions. A maximum of 3 missed cleavages were allowed and the minimum peptide length was set to six amino acids. The ptmRS algorithm was used for scoring phosphorylation sites considering site probability threshold >75. The filter High Confidence (FDR q value = 0.01) was applied on both peptide and protein levels.

Lysine acetylation identification and stoichiometry calculations were performed with the Pview software (28). For peptide identification, the maximum tolerance was set to 10 ppm for precursor and 0.02 Da for fragment ions, and up to 1% FDR was allowed. For the stoichiometry calculations, the isotopic tolerance was set to 3.5 ppm and at least four peaks in the XIC, allowing only one missing point in between. The MS proteomic data have been deposited to the ProteomeXchange Consortium *via* the PRIDE (29) partner repository with the data set identifier PXD027173.

### 2.7 Statistics and Bioinformatics

Pre-processing was performed for each brain region individually within the Perseus software (version 1.6.2.3) (30) both for global proteomics and phosphoproteomics. Technical replicates from phosphoproteomic analysis were averaged by taking their median. For qualitative comparison of the disease groups, we considered proteins, phosphopeptides and acetylated peptides that have at least 70% valid values in one and less than 10% valid values in the other condition (AD and control). For quantitative analysis, proteins and phosphopeptides with minimum 66% valid values in each disease group (AD and control), plus “on-off-like” proteins (less than 33% valid values in one group and at least 66% valid values in the other) were kept for further processing. Intensities were log2-transformed and median normalized, followed by replacing the missing values from a normal distribution (width = 0.3, downshift = 1.8). The phosphorylation state of proteins was calculated based on phosphopeptide intensities corrected for total protein abundance.

Further statistical analyses were conducted in R (version 4.0). Sex, age and postmortem intervals (PMI) were compared between the groups (AD and control) in each region using Fisher’s exact test (for binary data) and Wilcoxon rank sum test (for continuous numerical values) to determine whether these variables differ between groups. The tests showed that the disease groups were unbalanced with respect to age, a tendency for higher age in AD group was observable in HP and CA1, and the Wilcoxon rank sum test was significant in EC and PRC (**Figure S1**). Linear regression analysis was performed to address the proteomic, phosphoproteomic and phosphorylation status-related changes associated with disease category. Proteins and phosphopeptides with p-value < 0.05 and log2 fold change ≥ |0.7| were considered statistically significant. To filter out the effect of sex, age and PMI in each brain region, proteins and phosphopeptides that were significantly affected by these covariates (linear regression analysis, p-value < 0.05) were excluded from the biological interpretation of the results. Data derived from the global proteome and phosphoproteome analyses were combined to detect the kinases present in our dataset.

To follow AD progression through the comparison of different brain regions, log2-intensities of each protein and phosphopeptide in the AD samples were subtracted by the median log2-intensity of the same protein and phosphopeptide in the control samples. These log2 fold change values were then compared across regions using an ANOVA test followed by a Tukey HSD post-hoc test. Proteins and phosphopeptides with significant ANOVA p-value (p-value < 0.05) were then subjected to hierarchical clustering (complete linkage, Euclidean distance). The tree cutting was performed *via* the cutreeDynamic function from the dynamicTreeCut R package (version 1.63-1) with the following settings: minClusterSize = 100, method = hybrid, deepSplit = 1. Furthermore, pre-ranked Gene Set Enrichment Analysis *via* the clusterProfiler R package (*vs*. 3.18.1) (31) was performed on the log2 fold change differences across the regions, utilizing the Hallmark (32) and the ImmuneSigDB gene sets (33).

Data pre-processing for the acetyl stoichiometry analysis was performed in Excel and in R. The stoichiometry calculations were performed as reported previously (34). Acetylation ratios were calculated by taking the sum of endogenously acetylated peptides’ intensity divided by the sum of all peptides’ intensity (sum of endogenously and chemically acetylated). For comparison of the peptides’ acetylation ratio between disease groups, a Wilcoxon test was applied on peptides with at least 40% valid values in each disease group. Spatial progression of the brain areas was not addressed at the acetylation level due to the high percentage of missing values.

Additional bioinformatics analyses were performed using Reactome (35), and visualizations were done utilizing Cytoscape (36), Coral (37), and KEGG mapper (38).

## 3 Results

### 3.1 Protein Profiles of AD Vulnerable Brain Areas During the Limbic Stage

**Figure 1:**
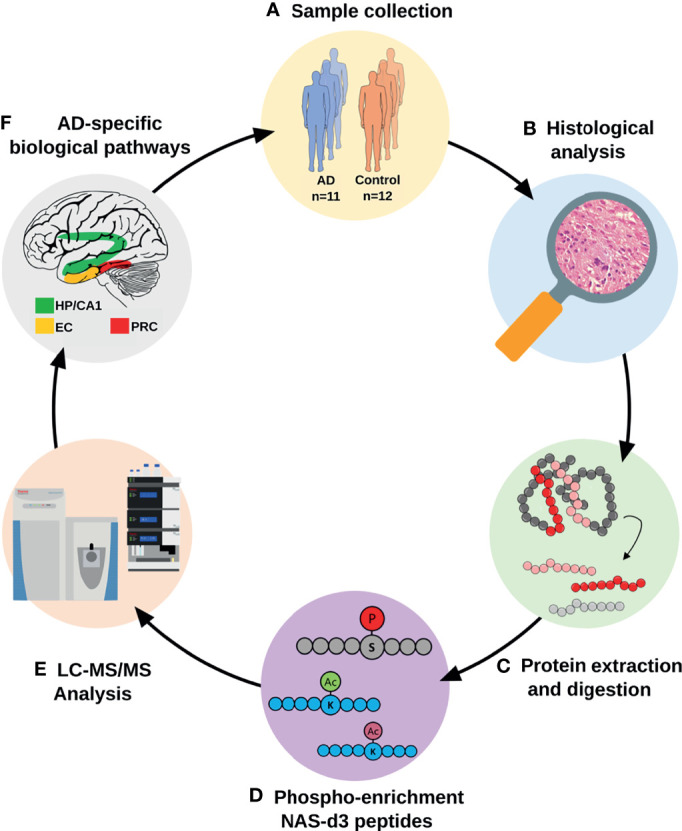
Analytical workflow for the proteomic characterization of AD vulnerable brain areas. AD and control tissues from four different brain areas were isolated after histological evaluation. Proteins were extracted and subjected to a proteomics and bioinformatics workflow, including the characterization of changes in protein acetylation and phosphorylation patterns. (AD, Alzheimer’s disease; CA1, CA1 region of the hippocampal complex; EC, Entorhinal cortex; HP, Hippocampus; LC-MS/MS, liquid chromatography-tandem mass spectrometry; NAS-d3, N-acetoxy-succinimide-d3; PRC, Perirhinal cortex).

To dissect the proteomic profiles of AD vulnerable brain areas in the limbic stage, we developed an analytical workflow (**Figure 1**) that integrates global and phosphoproteome analysis, including analysis of both phosphopeptide abundance and phosphorylation status of peptides (see *Methods* section), and acetylation stoichiometry of peptides. **Table 1** summarizes the total number of identified and quantified protein groups, phosphopeptides and acetylated peptides derived from global-, phosphoproteome and acetylome analyses, respectively in each AD-brain area.

**Table 1:** Quantitative summary of the global-, phosphoproteome and acetylome analyses in the four studied AD-brain areas (HP, hippocampus; CA1, CA1 region of the hippocampal cortex; EC, entorhinal cortex; PRC, perirhinal cortex).

Brain area	Global proteome – proteins					Phosphoproteome - phosphopeptides					Phosphoproteome – phosphorylation state			Acetylome – acetylated peptides				
	Total*	ON in AD	OFF in AD	UP-regulated in AD	DOWN-regulated in AD	Total*	ON in AD	OFF in AD	UP-regulated in AD	DOWN-regulated in AD	Total*	UP-regulated in AD	DOWN-regulated in AD	Total*	ON in AD	OFF in AD	UP-regulated in AD	DOWN-regulated in AD
**HP**	5411	58	11	52	63	8029	293	94	444	237	6336	327	166	186	18	19	1	1
**CA1**	5556	27	9	21	45	7207	203	15	75	27	5828	48	24	217	1	7	2	2
**EC**	4946	6	10	30	33	9166	12	8	18	55	7059	12	20	104	–	2	3	6
**PRC**	5486	6	8	4	14	8134	21	56	51	37	6490	26	29	155	2	1	3	4

^*^Total number of proteins, phosphopeptides and acetylated peptides used for quantitative analysis.

When comparing disease groups, we first focused on qualitative analysis of the data (see *Methods* section) considering the proteins predominantly represented in one condition (i.e., on-off proteins), and then performed quantitative analysis of each region and dissection of spatial progression of AD across brain vulnerable areas.

#### 3.1.1 Hippocampus

**Figure 2:**
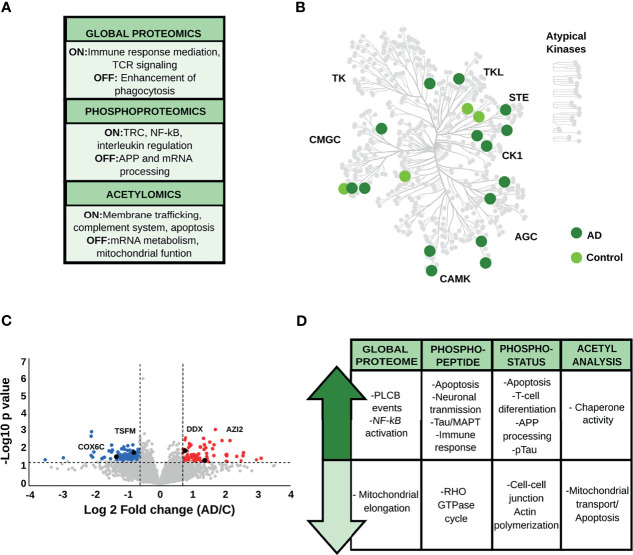
**(A)** Main biological processes representative for the hippocampus (HP) in the qualitative analysis. The “ON” condition represents biological processes and pathways that are associated with AD samples. On the contrary, the “OFF” condition represents biological processes and pathways that are linked to control samples. **(B)** Kinome analysis of the HP based on the qualitative comparison of AD and control samples. **(C)** Volcano plot derived from the global proteome data, as an example of the variation in the relative abundances of proteins between AD and control samples. **(D)** Relevant biological processes and pathways dysregulated in the HP across all datasets.

Qualitative analysis of the global proteome data (**Supplementary Table S2**) revealed the overrepresentation of proteins linked to immune mediation and T-cell receptor (TCR) signaling in AD patients, while those restricted to control brains were involved in the enhancement of phagocytosis in monocytes and macrophages. Phosphopeptides exclusively identified in AD are also linked to TCR-receptor activation and crucial pro-inflammatory pathways such as NF-κB activation and interleukins regulation. Moreover, phosphopeptides identified only in control brains are related to APP processing and mRNA metabolism (**Figure 2A** and **Supplementary Table S2**). The hippocampal kinome map (**Figure 2B**) detected differences in the presence of specific kinase families between AD and control conditions. For example, CDK16, a kinase that regulates the vesicle-mediated transport processes and exocytosis was found exclusively in control samples, whereas kinases involved in the microtubule-associated protein tau (Tau) phosphorylation, such as serine/threonine-protein kinase MARK2 and tau-tubulin kinase 1 were predominant in AD samples (**SupplementaryTable S2**). In addition, AD-brains showed a predominance of acetylated peptides involved in membrane trafficking, complement system activation, and apoptosis, while control condition exhibited acetylated peptides related to mitochondrial function and mRNA metabolism (**Supplementary Table S2**).

Regarding the quantitative analysis of the global proteome data (**Figures 2C, D** and **Supplementary Table S3**), down-regulated proteins in AD were mainly linked to mitochondrial function and cell-cell adhesion. AD brains displayed increased abundance of proteins associated with PLCB events (e.g., inositol 1,4,5-trisphosphate receptor type 1, and adenylate cyclase type 9) and pro-inflammatory activity such as the activation of complement cascade and the NF-κB pathway (e.g., plasminogen, protein kinase C delta type, ATP-dependent RNA helicase DDX1, and 5-azacytidine-induced protein 2).

Quantitative phosphopeptide analysis (**Figure 2D**, **Supplementary Table S4**) detected a decrease in abundances of phosphopeptides involved in the Rho GTPase cycle, such as protein scribble homolog and protein-tyrosine kinase 2-beta in AD brains. In contrast, phosphopeptides that showed increased levels in AD condition are mainly involved in neuronal transmission, apoptosis, and immune activation (e.g., 3-phosphoinositide-dependent protein kinase 1, Rho-related GTP-binding protein RhoG, and the COP9 signalosome complex subunit). More importantly, we found proteins related to Tau hyperphosphorylation, such as dedicator of cytokinesis protein 3, previously proposed as a key protein for AD development (39). Concerning the phosphorylation status analysis (**Supplementary Table S5**), we found that signaling pathways related to cell-cell communication and actin polymerization, represented by proteins such as neural Wiskott-Aldrich syndrome protein and protein-tyrosine kinase 2-beta, were down-regulated in AD. In addition, phosphoproteins involved in apoptosis (e.g., apoptotic chromatin condensation inducer in the nucleus), T-cell activation (e.g., drebrin-like protein, tyrosine-protein kinase Yes, raftlin), and Th1 differentiation (e.g., semaphorin-4A) were up-regulated in AD. In parallel, we observed an increase in classical AD hallmarks such as the hyperphosphorylated Tau (pTau), and proteins involved in APP processing, such as the Myc box-dependent-interacting protein 1. Acetyl stoichiometry analysis detected a decrease in the acetylation of proteins linked to mitochondrial transport and apoptosis, such as DnaJ homolog subfamily A member 1, and an increase in the acetylation of chaperone proteins such as heat shock protein HSP 90-alpha (**Figure 2D** and **Supplementary Table S6**).

Taken together, the integration of proteomic data, including the PTM analyses, suggests that the HP area has a biological signature characterized by an increase of the pro-inflammatory response. Activation of T-cell receptors and differentiation to a Th1-type immune response appears to be one of the main features of the proteome profile in the hippocampus during the limbic stage of AD. Particularly, we also observed alterations in classical AD-hallmarks related to pTau and APP processing.

#### 3.1.2 CA1 Region of the Hippocampal Complex

**Figure 3:**
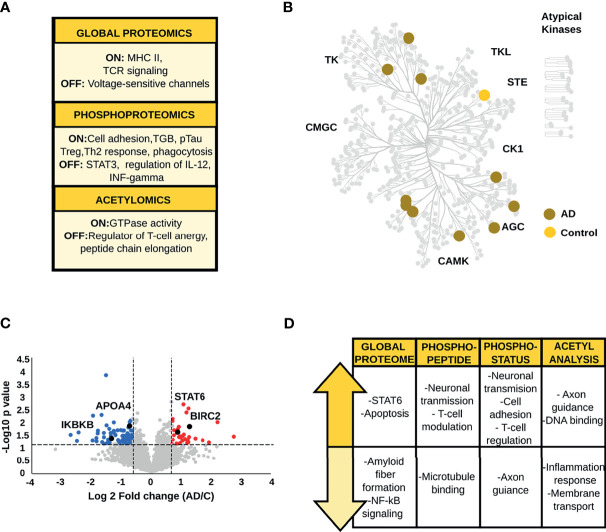
**(A)** Biological processes emerging from the qualitative analysis of the CA1 region. **(B)** Kinome map of the CA1 region derived from the qualitative analysis of AD and control samples **(C)** Volcano plot of the global proteome data representing the variation in protein abundances between AD and control samples. **(D)** Main biological processes and pathways altered in the CA1 region.

The regulation of membrane trafficking, the expression of TCR receptors and major histocompatibility complex II (MHC-II) are the major biological signatures of proteins found exclusively in AD brains in the qualitative global proteome analysis (**Figure 3A** and **Supplementary Table S2**). On the other hand, proteins identified only in control brains regulate voltage-sensitive channels. Qualitative phosphopeptide analysis and CA1 kinome mapping revealed the presence of proteins related to the immune system, neurotransmission and neural cell adhesion in AD condition (**Figures 3A, B** and **Supplementary Table S2**). Phosphopeptides and kinases restricted to control brains (e.g., mitogen-activated protein kinase 5) mediate the transduction of inflammatory and stress-activated signal regulation, such as the tumor necrosis factor (TNF) (**Figures 3A, B** and **Supplementary Table S2**). Finally, the srGAP3 protein, mainly associated with GTPase activity was only found to be endogenously acetylated in AD patients. On the contrary, acetylated peptides found exclusively in control brains have a critical role in regulating T-cell anergy and peptide chain elongation (**Supplementary Table S2**).

The analysis of global protein changes revealed lower expression levels of proteins that regulate amyloid fiber formation such as APOA4, and immune related proteins such as the inhibitor of nuclear factor kappa-B kinase subunit beta in AD. In contrast, up-regulated proteins in AD brains, such as baculoviral IAP repeat-containing protein 2, baculoviral IAP repeat-containing protein 6 and ADP-ribosylation factor-like protein 2 are related to the modulation of apoptosis and inflammatory signaling (**Figures 3C, D** and **Supplementary Table S3**). More importantly, one of the main findings in AD brains was the increased level of STAT6 a crucial player in the activation of the anti-inflammatory Th2/M2 profile (40).

Phosphoproteome analysis and acetyl-stoichiometry data showed protein alterations in congruence with the biological changes observed in the global proteome (**Figure 3D** and **Supplementary Tables S3**–**6**). The phosphopeptides and phosphosites down-regulated in AD are primarily linked to the stability of microtubule-associated proteins and axon guidance (e.g., ankyrin-2 and ephrin type-A receptor 5). Additionally, phosphopeptides and phosphosites with higher abundances in AD compared to control patients were related to glutamatergic neurotransmission (e.g., calcium/calmodulin-dependent protein kinase type II subunit alpha, glutamate receptor-interacting protein 1, and glutamate receptor ionotropic, NMDA 2B). Also, negative regulation of T-cells and the suppression of type I interferon (IFN) production were essential findings, represented by alteration of proteins such as homer protein homolog and OTU domain-containing protein 5. Likewise, the acetyl-stoichiometry data showed that proteins involved in the inflammatory response and negative regulation of ion transmembrane transport were less acetylated in AD samples. Conversely, the degree of acetylation was higher in proteins whose function is linked to cytoskeletal membrane and axon guidance.

In summary, our data show an anti-inflammatory profile in the CA1 region. This brain area stands out by the negative regulation of T-cell activation and the inhibition of pro-inflammatory pathways such as NF-κB. The immune response exhibits crucial proteins involved in the alternative polarization to Th2/M2 phenotype. Last but not least, we observed a sustained increase in Tau phosphorylation and a decrease in APOA4 abundance, which are crucial events for AD-risk and development.

#### 3.1.3 Entorhinal Cortex

**Figure 4:**
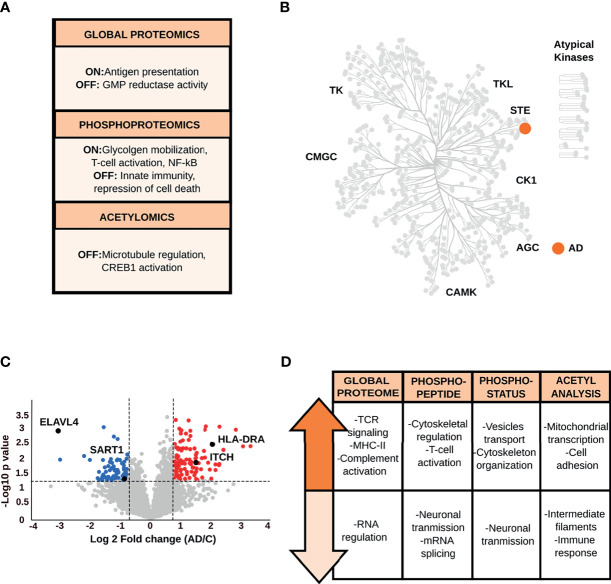
**(A)** Main biological processes derived from the qualitative analysis of the EC. **(B)** Kinome analysis of the EC based on the qualitative comparison of AD and control data sets. **(C)** Volcano plot of the global proteome data representing the variation in protein abundances between AD and control samples in the EC. **(D)** Biological processes and pathways associated with the dysregulated proteins in the EC across all datasets.

The global proteome data analysis identified the guanosine monophosphate reductase 1 exclusively in control condition (**Figure 4A** and **Supplementary Table S2**). Qualitative phosphopeptide analysis revealed that phosphopeptides exclusively detected in AD brains are associated with glycogen mobilization, T-cell activation, and positive regulation of NF-κB signaling. In contrast, phosphopeptides present only in control samples, belong to proteins involved in innate immunity and transcriptional repression of cell death (**Supplementary Table S2**). Kinome characterization (**Figure 4B** and **Supplementary Table S2**) showed kinases required for the response to environmental stress and cytokines (e.g., mitogen-activated protein kinase 4) only in AD samples. The acetyl analysis detected acetylated peptides specific to controls, which are derived from proteins that participate in microtubule regulation and CREB activation.

Quantitative global proteome analysis showed the down-regulation of proteins (**Figure 4C** and **Supplementary Table S3**) related to neurotransmission and mRNA splicing and processing (e.g., U4/U6.U5 tri-snRNP-associated protein 1, ELAV-like protein 4). Proteins with increased levels in AD condition are mainly linked to TCR-activation and NF-κB-mediated transcription, such as F-box/WD repeat-containing protein 1A and receptor-type tyrosine-protein phosphatase C. However, another set of proteins, such as protein arginine N-methyltransferase 2, involved in NF-κB inhibition were also up-regulated in AD. Crucial immune proteins, such as MHC-II, complement C1s subcomponent were altered in the EC as well. The increased level of proteins (e.g., annexin A1, E3 ubiquitin-protein ligase Itchy homolog) that intervene in the immunomodulation of the T-cell differentiation into Th1 cells and negatively regulate differentiation into Th2 cells was also prominent in the EC (41, 42).

Quantitative changes at phosphorylation and acetylation levels (**Figure 4D** and **Supplementary Tables S4**–**6**) are closely related to the alterations in the global proteome. The phosphopeptide and phosphosite analyses revealed that biological processes such as neurotransmission (e.g., glutamine synthetase, glutamate ionotropic receptor) and mRNA splicing (e.g., U4/U6.U5 tri-snRNP-associated protein 2), including a specific repressor of the MAPT/Tau exon 10 splicing (e.g., splicing factor U2AF 65 kDa subunit) are down-regulated in AD. The increase in phosphopeptide and phosphorylation levels related to membrane-cytoskeleton-associated proteins (e.g., alpha-adducin), T-cell activation (e.g., receptor-type tyrosine-protein phosphatase C), vesicle transport (e.g., Rab-11B), and the glial fibrillary acidic protein were common features in the EC. Moreover, acetyl data reported lower acetylation stoichiometry in the glial fibrillary acidic protein and annexin A1 in AD brains, while an increase in the acetylation status of proteins involved in the regulation of mitochondrial transcription and cell adhesion (e.g., ADAM 23).

Our data display hints of evidence about the complexity of immunomodulation in the EC. Protein changes exhibit a hybrid immune profile that converges in the activation/inhibition of inflammatory pathways such as NF-κB and the modulation of the Th2-type response to a Th1 profile. Likewise, astroglial and inflammatory markers, such as glial fibrillary acidic protein (43), were found altered in their phosphorylation and/or acetylation state.

#### 3.1.4 Perirhinal Cortex

**Figure 5:**
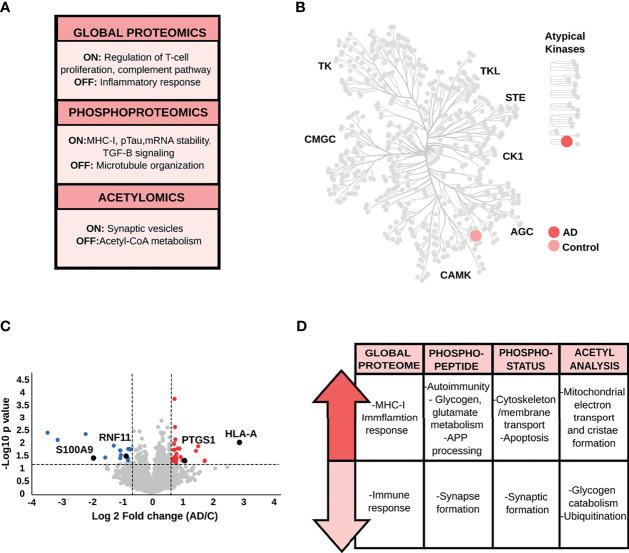
**(A)** Biological processes derived from the qualitative analysis of the PRC. **(B)** Kinome map of the PRC based on the qualitative comparison of AD and control samples. **(C)** Volcano plot of the PRC global proteome data, as an example of the variation in the relative abundances of proteins between AD and control samples. **(D)** Key biological processes and pathways represented by proteins altered between control and AD samples in the PRC.

Proteins exclusively identified in control samples are involved in inflammatory response (e.g., prostaglandin G/H synthase 1), while AD-specific proteins are involved in the negative regulation of T-cell proliferation and complement pathway (e.g., V-set and immunoglobulin domain-containing protein 4) (**Figure 5A** and **Supplementary Table S2**). Qualitative phosphoproteome analysis (**Figure 5A** and **Supplementary Table S2**), showed unique phosphopeptides in control samples, corresponding to proteins mainly associated with microtubule organization (e.g., centrosomal protein of 170 kDa). In parallel with these findings, we found phosphopeptides restricted to AD brains that correspond to the HLA class I histocompatibility antigen A alpha chain (MHC-I), Tau and TGF-beta signaling (e.g., E3 ubiquitin-protein ligase TRIM33). Mapped kinases absent in AD are related to signal transduction (e.g., serine/threonine-protein kinase DCLK2). In contrast, kinases specific to AD brains, such as E3 ubiquitin-protein ligase TRIM33, participate in the TGF-β signaling pathway (**Figure 5B** and **Supplementary Table S2**). Qualitative acetyl analysis revealed one acetylated peptide specific for control samples, which is located in the pyruvate dehydrogenase E1 component alpha subunit and linked to acetyl-CoA metabolism. The acetylated peptides restricted to AD condition originated from proteins involved in synaptic vesicles trafficking.

Quantitative analysis of PRC data resulted in down-regulated proteins, such as RING finger protein 11 ubiquitin-editing protein complex, that guarantees the provisional nature of inflammatory signaling pathways. Furthermore, we also found up-regulated proteins in AD patients, such as MHC-I, with a crucial role in the immune response (**Figures 5C, D** and **Supplementary Table S3**).

Quantitative phosphoproteome analysis detected a decrease in the level of phosphopeptides and phosphorylation status of proteins involved in synapsis formation (e.g., disks large-associated protein 4, Shank3), cytoskeleton organization and stability (e.g., serine/threonine-protein kinase TAO1, EMK-1), and chemokine receptor CXCR4 and CXCL12-induced cell chemotaxis (e.g., ubiquitin carboxyl-terminal hydrolase 14) (**Figure 5D** and **Supplementary Table S4–5**). Our statistical analysis uncovered up-regulated phosphopeptides representing proteins involved in preventing the aggregation of misfolded proteins (e.g., large proline-rich protein BAG6) and controlling the proteolytic processing of APP (e.g., transmembrane and coiled-coil domains protein 2). We also found proteins linked to microtubule-associated protein tau phosphorylation (e.g., MAP/microtubule affinity-regulating kinase 4) and glutamate and glycogen metabolism (e.g., glutamate dehydrogenase, glycogen phosphorylase). Lastly, we detected the increment of phosphorylated proteins related to T lymphocyte response (e.g., Rho family-interacting cell polarization regulator 2) and the prevention of the cell-intrinsic initiation of autoimmunity (e.g., three-prime repair exonuclease 1). On the other hand, phosphosites, which exhibited an increased level of phosphorylation in AD condition were mainly linked to cytoskeletal organization and ubiquitination (e.g., E3 ubiquitin-protein ligase UBR4), apoptosis mediation and stress fiber dissolution (e.g., STE20-like kinase), lysosome movement and stabilization of endoplasmic reticulum (e.g., BLOC-1-related complex subunit 5 and reticulon-4). The acetyl stoichiometry analysis found proteins with reduced level of acetylation, such as glycogen phosphorylase and the ubiquitin-conjugating enzyme E2 L3, principally related to glycogen metabolism and ubiquitination. On the contrary, most of the proteins showed higher level of acetylation in AD brains compared to controls are required for mitochondrial transport and morphology (e.g., MICOS complex subunit MIC19, Complex I-39kD) (**Figure 5D** and **Supplementary Table S6**).

In conclusion, the PRC proteome profile is mainly characterized by the prevention and processing of key AD-protein markers such as APP and Tau. This brain area is characterized by the potentiation in the presentation of endogenous antigens. Moreover, we observed an imbalance of key proteins that modulate the immune response to promote self-tolerance against a possible generation of self-antigens.

### 3.2 Dissecting the Molecular Trajectories Across the AD Vulnerable Areas During the Limbic Stage

The global proteome and PTM analysis conducted so far assessed the individual characteristics of each brain area vulnerable to AD, comparing the imbalances in protein expression and PTM status between AD and control cases. Our data revealed the predominant dysregulation of biological pathways related to the immune response, APP processing, Tau phosphorylation, cytoskeleton remodeling, and mRNA processing. These protein changes have a unique biological signature in each AD brain region. However, to provide a deep understanding of the brain region vulnerability in AD, we focused on comparing the fold changes/ratios (AD *vs*. control) in protein and phosphopeptide abundances, as well as in phosphorylation and acetylation status of the proteins/peptides identified across all brain areas. In this analysis, changes from HP to CA1 followed by EC and PRC were revealed. This approach allows us to find molecular trajectories shaped by alterations in protein abundances and variations in their PTM status that are potentially associated with the spatial progression of the disease.

**Figure 6:**
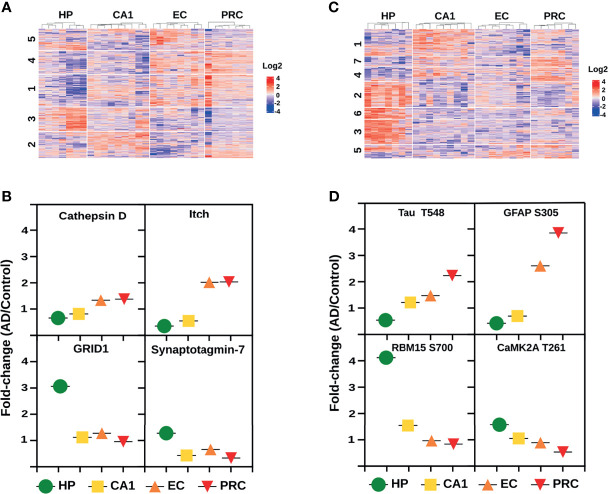
Molecular trajectories across the AD vulnerable areas. **(A)** Protein clusters from the global proteome analysis with altered log2 fold change (AD/C median) profiles across the four brain areas. **(B)** Log2 fold change profiles with increasing and decreasing tendencies across the four AD vulnerable brain areas of 4 representative proteins. Data derived from the global proteome analysis. **(C)** Phosphopeptide clusters with altered log2 fold changes (AD/C median) across the four brain areas. **(D)** Log2 fold change profiles with increasing and decreasing tendencies across the four AD vulnerable brain areas of 4 representative phosphoproteins. Data derived from the phosphoproteomic analysis.

Cluster analysis of the global proteome data (**Figure 6A** and **Supplementary Table S7**) showed 121 proteins with an increased expression profile, mainly localized in clusters 1 and 4 (**Figure 6A**). Cluster 1 revealed a gradual increase in protein expression primarily related to acetyl-CoA and amino acid metabolism (e.g., protein JTV-1, glycine amidinotransferase, and the mitochondrial 2-oxoisovalerate dehydrogenase subunit beta), especially serine metabolism (e.g., 3-phosphoglycerate dehydrogenase, cytoplasmic serine-tRNA ligase). In the same cluster, we observed proteins, such as hnRNPD0, involved in mRNA processing. The progressive increase of critical regulators of the immune response, such as E3 ubiquitin-protein ligase Itchy homolog (**Figure 6B**), a key regulator of Th2, as well as proteins involved in T-cell receptor activation, NF-κB pathway (e.g., core-binding factor subunit beta, pirin), “eat-me” signals [e.g., multiple epidermal growth factor-like domains protein 10 (MEGF10)], and microglia/macrophages markers (e.g., monocyte differentiation antigen CD14) were also identified. Cluster 4 was characterized by increasing levels of proteins linked to PI3K/AKT signaling (e.g., GRB2-associated-binding protein 2, fibroblast growth factor receptor 3), metabolism (e.g., glutamate dehydrogenase, nicotinamide phosphoribosyltransferase, fatty-acid amide hydrolase), and APP processing such as cathepsin D (**Figure 6B**). In addition, proteins related to the innate and adaptive immune response (e.g., butyrophilin subfamily 3 member A3, multiple EGF-like domains protein 10) and antigen presentation (MHC class II antigen DRA) were also found in the same protein cluster. Lastly, our analysis uncovered 77 proteins with decreasing expression profile, most of them grouped in cluster 3. These proteins are mainly involved in synaptic function (e.g., glutamate ionotropic receptor, gamma-aminobutyric acid receptor, synaptotagmin-7) (**Figure 6B**), and in the co-activation of the transcriptional activities of several nuclear receptors such as PPARG (e.g., nuclear receptor coactivator 7), an essential promoter of the sustained activation of the M2 anti-inflammatory profile (44).

Phosphoproteome analysis revealed a progressive increase in the phosphopeptide abundances and phosphorylation status of 82 and 60 proteins, and a decrease of 167 and 119 proteins, respectively. Phosphorylation status analysis displayed 100% overlap with the phosphopeptide abundance data (**Supplementary Table S7**). Protein clusters with increased phosphorylation profiles are mostly distributed in three clusters, i.e., clusters 4, 5 and 7 (**Figure 6C**). Cluster 4 includes proteins that are associated with pre-mRNA processing events (e.g., SRm160, CD2 tail-binding protein 2), positive regulation of NF-κB signaling (SAM and SH3 domain-containing protein 1) and maintaining immune self-tolerance (e.g., SAPS domain family member 3). Cluster 5 is characterized by oxidative deamination (e.g., amine oxidase), cytoskeleton regulation (e.g., neurofilament medium polypeptide, filamin-A, amphiphysin II), T-cell activation (e.g., leukocyte common antigen) and the increment of CD45, a hematopoietic cell marker (45). In cluster 7, we found proteins involved in the Rho GTPases pathway (e.g., PH-interacting protein, Rho GTPase-activating protein 44) and the TGF-beta signaling pathway modulation (e.g., protein PML). We also detected a continuous increase in the phosphorylation status of well-established AD markers such as Tau and glial fibrillary acidic protein (**Figure 6D**) across the four brain regions. Proteins from clusters 2, 3 and 6 showed decreasing phosphorylation profiles (**Figure 6C**). Cluster 2 is composed of proteins from the CaMK family (**Figure 6D**) that mediates the phosphorylation of CREB (e.g., CAMK2A, CAMK2D, CAMK2B), mTOR signaling (e.g., S6K-beta-1), and negative regulation of NF-κB (e.g., hKSR2). Vesicle transport (e.g., clathrin heavy chain, DNAJC6), ubiquitination (e.g., E3 ubiquitin-protein ligase HUWE1, E3 ubiquitin-protein ligase RNF31), modulation of Wnt signaling (e.g., cyclin-dependent kinase 14), alternative splicing of mRNAs by proteins such as RNA-binding protein 15 (**Figure 6D**), are the main biological pathways related to cluster 3. Finally, cluster 6 is represented by proteins that modulate the actin cytoskeleton (e.g., Abl interactor 2), APP processing (e.g., amyloid-beta A4 precursor protein-binding family A member 2), and mRNA metabolism (e.g., Pumilio homolog 2, putative RNA-binding protein 15B).

### 3.3 Antimicrobial Peptides During the Limbic Stage of AD

**Figure 7:**
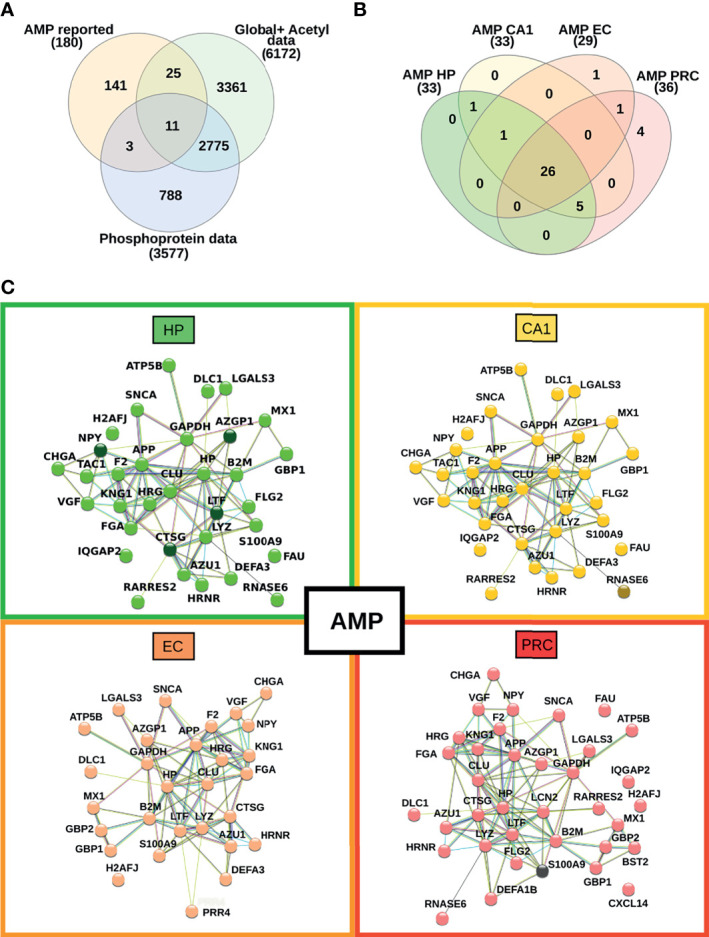
Mapping of proteins with antimicrobial peptide function across the four brain regions. **(A)** Venn diagram representing the overlap between our current proteomic data set and proteins with previously reported antimicrobial peptide activity. **(B)** Venn diagram showing the overlap of AMPs across all brain areas in this study. **(C)** Interaction network between the identified AMPs in each investigated brain area. Nodes highlighted with darker colors represent proteins with changes in their relative abundance.

The proteomic profiles of vulnerable brain areas during the AD limbic stage revealed an imbalance of proteins related to the immune response. Currently, the hypothesis that links microbial insults to immune system activation in AD, which ultimately leads to the production of antimicrobial peptides (AMPs), has been strengthened (46). To explore the distribution of proteins with antimicrobial activities across the four brain regions and their changes in expression and/or post-translational modification status, we compared our complete proteomic data set with the recently published UDAMP database (47). A total of 39 AMPs were detected, 26 of which were found in all brain regions (**Figure 7A**). The HP and CA1 regions display the same AMP profiles while EC and PRC exhibited exclusive proteins with AMP functions (e.g., proline-rich protein 4 in EC, and neutrophil defensin 1, C-X-C motif chemokine 14, bone marrow stromal antigen 2 and neutrophil gelatinase-associated lipocalin in PRC) (**Figure 7B**). Despite this overlap, the data shows differences in changes in their relative abundance when comparing AD with controls across different brain areas.

**Figure 7C** highlights the interaction networks and dysregulation pattern of proteins with AMP function in each vulnerable region of AD. In particular, the most prominent alterations occur in brain areas with pronounced pro-inflammatory proteomic profiles, such as HP. Three AMP proteins showed increased abundances in HP, such as lactotransferrin, cathepsin G, and zinc-alpha-2-glycoprotein. Also, in HP the phosphorylation status of the pro-neuropeptide Y stands out in AD samples compared to controls. The phosphorylated form of ribonuclease K6 was found exclusively in AD samples from the CA1 region, but not in controls. Conversely, no significant changes in phosphorylation level or global protein expression were observed between AD and controls in EC. A decrease in the abundance of protein S100-A9 was observed in AD samples from the PRC. Taken together, the data reveals that proteins with reported AMP functions are similarly distributed in different vulnerable AD areas in the limbic stage of the disease, but the changes in their abundances are more evident in the hippocampus.

## 4 Discussion

Amyloid deposition and neurofibrillary degeneration in AD gradually affect the central nervous system in a specific pattern, but the molecular mechanisms behind the vulnerability of individual brain areas to the disease remain unclear. Here, we conducted a proteomic study that integrated additional dimensions, such as the phosphorylation and acetylation status of proteins to investigate the evolution of protein profiles and the possible impact of these PTMs in brain regions affected during the limbic stage of AD.

Our data revealed regional heterogeneity of the proteome profiles in some brain areas affected in AD, highlighting the alteration of proteins related to the immune system. It is well-known that microglia acquire region-specific gene expression profiles that provide them with special responsiveness during health and disease in different brain regions, which underlie their functional contribution to disease development (48, 49). Here, we found protein profiles that reflect the unique immune response of each AD-brain region. For instance, the main feature of the hippocampus is an increased pro-inflammatory response. Multi-omics analysis suggests the activation of important pathways such as NF-κB and proteins related to the activation of T-cell receptors and Th1 response. Previous studies demonstrated the complex dynamics of the immune response during AD and a significant contribution to the exacerbation of neurodegeneration (8, 50–52) proposing the activation of the NF-κB pathway as one of the earliest inflammatory hallmarks in AD (53).

Proteomic analysis of the CA1 region of the hippocampal complex showed an anti-inflammatory profile in this region. CA1 region is characterized by the negative regulation of T-cell activation and the inhibition of pro-inflammatory pathways such as NF-κB. A large-scale proteomic study observed a significant increase in markers associated with an anti-inflammatory state of the microglia and astrocyte in the dorsolateral prefrontal cortex of AD patients (20). Our data suggest a similar profile in the CA1 region during the limbic stage, with an increase in key protein levels such as STAT6 involved in the alternative polarization of the Th2/M2 phenotype (54, 55). The Th2/M2 immune phenotype has been associated with a higher capacity of Aβ clearance by microglia and tissue repair (56, 57). In parallel, other findings also indicate that the M2 phenotype in the hippocampus of APP transgenic TgCRND8 mice leads to the exacerbation of amyloid deposition *in vivo* when is induced by a short-term mIL-4 expression (58).

Evidence of the imbalance in the pro- and anti-inflammatory response is even more complex in the entorhinal cortex. As mentioned, our data displayed proteomic changes that converge in the activation/inhibition of inflammatory pathways such as NF-κB, the up-regulation of MHC-II, and the transition from a Th2-type response to a Th1 profile. Jimenez and colleagues (59) reported a dichotomy of the inflammatory responses in the hippocampus, presenting evidence for a change in the activation pattern of microglia during AD progression. Their results suggest that the microglia switches from an activated phenotype to an inflammatory profile at the onset of the disease (59). However, in advanced stages, the microglia return to a classical phenotype. The hybrid immune profile observed in our proteomic data might partially reflect the spatial progression of the disease in EC.

The dysregulation of proteins involved in antigenic presentation and self-tolerance is one of the main molecular signatures of the perirhinal cortex. The up-regulation of the MHC-I was an important finding in this brain area. Previously, increased levels of MHC class I molecules in vascular endothelial cells, and the expression of MHC-I and MHC-II by microglia have been detected in post-mortem brain tissues from the medial temporal cortex of AD patients (60). In addition, MHC-I expression in the endothelium was found to be associated with the infiltration of CD8+ T cells into the brain parenchyma (61). Similarly, CD8+ T infiltration (and CD4+ T) has been reported in AD as not fully differentiated effector cells (62). However, there is no solid evidence of cytotoxic activity of CD8+ T-cells in AD so far (63). Furthermore, our data suggest that a concomitant immunomodulatory response is driven by a decrease in the S100A9 level and an increase in proteins involved in the prevention of the cell-intrinsic initiation of autoimmunity, such as three-prime repair exonuclease 1 (64).

Despite the heterogeneous histological composition of the investigated brain areas affected in AD, one of our main findings is the detection of trends in protein abundance and their PTM status that might contribute to the spatial progression of AD. We found a sharper rise in classical hallmarks of AD progression, such as an increment in Tau phosphorylation (39) and the expression of proteins involved in APP processing such as cathepsin D (65) across the analysis of the four different brain areas. In conjunction with these changes, we also observed the alteration of proteins linked to mRNA processing, mTOR and Wnt signaling, cytoskeleton regulation, metabolism, and synaptic functions, as previously reported by similar studies (14, 15, 18).

It is noteworthy that we detected an interesting trend in protein expression and/or PTMs associated with the adaptive and innate immune response. For instance, we found the progressive increase of proteins linked to the antigenic presentation (MHC-II), positive regulation of NF-κB signaling (e.g., SAM and SH3 domain-containing protein 1), and critical inhibitors of the Th2/M2 profile (e.g., E3 ubiquitin-protein ligase Itchy homolog) together with the concomitant decrease in the expression of the coactivator of nuclear receptor PPARG (nuclear receptor coactivator 7). We also observed an increment in CD14 level, a key microglia/macrophage/dendritic marker that modulates microglial TLR4 activities (66), triggers the release of pro-inflammatory cytokines (67), and interferes with T-cell migration and function (68, 69). Additionally, up-regulation of CD45, a hematopoietic antigen that influences the phagocytic capacity of Aβ by the microglia, were found (45). Moreover, we hypothesize that the continued increase in MEGF10 across the brain areas might suggest that astrocytes and their phagocytic function play an essential role as a compensatory mechanism against microglial impairments previously reported in AD (70–72). Gradual changes in the expression of these immune-related proteins can lead to a sustained inflammatory status in AD. Chronic inflammation triggers plaque deposition and NFT accumulation, exacerbating the memory impairments in AD patients (73).

The analysis of proteins with potential AMP functions displays a similar distribution across the AD-brain areas. AMPs have a dual role related to pro-inflammation and immunomodulation (46, 47). Interestingly, the alteration in their abundance is more prominent in the HP, the brain region characterized by a pro-inflammatory profile. However, the specific contribution of AMPs to the control of the neuroinflammatory process in early affected AD-brain areas remains to be elucidated.

We are aware that the analysis of a restricted number of samples is a limitation of our study. However, our findings were consistent across the four brain areas studied. Further confirmatory studies will be necessary using a larger well-defined patient cohort to unravel the role of these immunological signatures in individual brain areas, and their impact on the development of AD. In conclusion, our data suggest that brain regions affected in AD present unique molecular signatures, but there are also spatially coordinated changes across brain areas that point to neuroinflammation as a major player in AD pathology.

## Data Availability Statement

The datasets presented in this study can be found in online repositories. The names of the repository/repositories and accession number(s) can be found in the article/**Supplementary Material**.

## Ethics Statement

The studies involving human participants were reviewed and approved by Regional and Institutional Committee of Science and Research Ethics of Scientific Council of Health, Budapest, Hungary. The patients/participants provided their written informed consent to participate in this study.

## Author Contributions

EV and MR conceived and planned the experimental design. PD was involved in the study design. TH performed the neuropathological analysis. MP dissected the post-mortem tissue samples and ER managed the dissection procedures. EV and BS performed the experiments, analyzed the data and performed the biological interpretation. JG, JR, and MR advised mass spectrometry analysis. EV, BS, and MR wrote the article. MR and GD supervised the work. All authors contributed to the article and approved the submitted version.

## Funding

This study was supported by the Hungarian National Brain Research Program (2017-1.2.1-NKP-2017-00002) for MP and ER; by FAPERJ (grant E 26/202.650/2018 and grant E-26/210.173/2018) and CNPq (grant 315167/2020-3, 440613/2016-7 and grant 308341-2019-8) for FN and GD. EV thanks the financial support from the Brazilian foundation CAPES (grant 88887.130697) and BS acknowledges support from the Erasmus+ and Campus Mundi Programme.

## Conflict of Interest

The authors declare that the research was conducted in the absence of any commercial or financial relationships that could be construed as a potential conflict of interest.

## Publisher’s Note

All claims expressed in this article are solely those of the authors and do not necessarily represent those of their affiliated organizations, or those of the publisher, the editors and the reviewers. Any product that may be evaluated in this article, or claim that may be made by its manufacturer, is not guaranteed or endorsed by the publisher.

## Supplementary Material

Supplementary Figure 1 Comparison of sex, age and postmortem intervals between the disease groups (control *vs* AD) in the 4 brain regions.

Supplementary Table 1 Characteristics of the patient cohort.

Supplementary Table 2 Summary of the qualitative analysis.

Supplementary Table 3 Summary of the global proteome analysis.

Supplementary Table 4 Summary of the phosphopeptide analysis.

Supplementary Table 5 Summary of the phosphorylation status analysis.

Supplementary Table 6 Summary of the acetylome analysis.

Supplementary Table 7 Comparison of the four vulnerable AD brain areas.
